# Platelet Concentrates in Tooth Replantation: A Narrative Review and Evidence Synthesis of Periodontal and Pulpal Healing

**DOI:** 10.1002/cre2.70507

**Published:** 2026-11-04

**Authors:** Walaa Almarji, Mohannad Laflouf

**Affiliations:** ^1^ Department of Pediatric Dentistry Damascus University Damascus Syria

**Keywords:** platelet‐rich fibrin, platelet‐rich plasma, root resorption, tooth avulsion

## Abstract

**Objectives:**

The prognosis of an avulsed, replanted tooth over the long term hinges on whether the periodontal ligament (PDL) and the dental pulp can be preserved or regenerated. When extra‐oral dry time is prolonged, PDL necrosis follows, and the root becomes prone to inflammatory or replacement (ankylotic) resorption. Platelet concentrates—platelet‐rich plasma (PRP), leukocyte‐ and platelet‐rich plasma (L‐PRP), and platelet‐rich fibrin (PRF)—are autologous biomaterials that supply growth factors and may influence this healing process. This review asks whether autologous platelet concentrates reduce inflammatory and replacement (ankylotic) root resorption and improve periodontal and pulpal healing after delayed or intentional tooth replantation, compared with no adjunct, and whether residual periodontal ligament (PDL) viability changes this effect.

**Material and Methods:**

This narrative synthesis (not a systematic review) draws together recent controlled clinical and animal studies with the case‐report and preclinical literature already captured in the only published systematic review on this topic. The search covered PubMed, Google Scholar, the Cochrane Library, Embase, Scopus, and Web of Science, from January to May 2026.

**Results:**

In preclinical models, L‐PRP and PRF promote pulpal repair, tertiary dentin formation, and attenuated inflammatory root resorption, although neither consistently prevents ankylosis once PDL viability has been lost. In one retrospective comparative study of intentional replantation in hopeless teeth, PRF combined with root‐surface biomodification produced greater clinical attachment gain and a larger reduction in probing depth than replantation alone, a direction consistent with earlier case reports. Small, largely non‐randomized samples and unstandardized protocols still limit this evidence.

**Conclusions:**

Platelet concentrates are biologically plausible adjuncts that improve periodontal and pulpal healing indices, yet they do not reliably prevent ankylosis. Their clinical role will remain undefined until prospective randomized trials with standardized protocols are available.

## 1 Introduction

Dentoalveolar trauma affecting the permanent dentition remains a major clinical problem in pediatric dentistry, endodontics, and dental traumatology (Kostka et al. 2014; Galler et al. 2021). Tooth avulsion—the complete displacement of a tooth from its alveolar socket—is among the most severe of these injuries, accounting for roughly 0.5% to 3% of all traumatic dental injuries to permanent teeth (Kostka et al. 2014; Galler et al. 2021). Managing an avulsed tooth means restoring its physiological attachment apparatus as quickly as possible, comprising the periodontal ligament (PDL), cementum, and alveolar bone (Kostka et al. 2014; Polimeni et al. 2006).

Two factors chiefly determine the long‐term prognosis of a replanted avulsed tooth: the total extra‐oral period and the storage conditions before replantation (Kostka et al. 2014; Galler et al. 2021). Once extra‐oral dry time passes 30–60 min, extra‐alveolar hypoxia and desiccation cause mass necrosis of the PDL cells still attached to the root surface (Galler et al. 2021; Polimeni et al. 2006). This non‐viable PDL matrix then provokes a marked foreign‐body inflammatory response on replantation, which frequently ends in one of two severe complications: inflammatory root resorption, driven by bacterial and necrotic breakdown products, or replacement resorption (ankylosis), in which osteoclasts and osteoblasts fuse bone directly to dentin without an intervening functional PDL space (Galler et al. 2021; Polimeni et al. 2006). Delayed replantation is a common clinical scenario rather than a rare one. Reported rates of inflammatory and replacement root resorption after delayed replantation reach as high as 23% and 51%, respectively. This points to the clinical value of adjunctive biological strategies able to modulate these processes favorably (Galler et al. 2021; Kohsar et al. 2022; Miron et al. 2026).

Interest in biological methods for modulating wound healing and encouraging PDL cell regeneration goes back more than three decades, starting with early observations that autologous plasma influenced periodontal healing of replanted teeth in animal models (Nasjleti et al. 1986). Autologous platelet concentrates, ranging from first‐generation platelet‐rich plasma (PRP) and leukocyte‐ and platelet‐rich plasma (L‐PRP) to second‐generation platelet‐rich fibrin (PRF) and its leukocyte‐ and platelet‐rich fibrin (L‐PRF) variant—by far the most widely used and extensively documented second‐generation formulation in current clinical practice—have since drawn wide interest in regenerative dentistry (Miron et al. 2026; Quirynen, Sculean, et al. 2025; Calciolari et al. 2025). A dedicated special issue of Periodontology 2000, published in 2025, has since consolidated the evidence base for autologous platelet concentrates across dental disciplines into evidence‐based clinical guidelines, underscoring how rapidly this literature continues to evolve (Quirynen, Sculean, et al. 2025, Quirynen, Blanco, et al. 2025). Rich in autologous growth factors, cytokines, and a physiological fibrin matrix, these biomaterials support cell migration, proliferation, angiogenesis, and matrix synthesis (Polimeni et al. 2006; Miron et al. 2026). The only systematic review focused specifically on their use in tooth replantation identified 10 case reports, three animal studies, one combined animal/in vitro study, and one purely in vitro study; this body of evidence is incorporated below alongside more recent controlled clinical and animal data (Khurshid et al. 2022).

Despite this growing body of preclinical and case‐level evidence, no narrative synthesis has yet brought the controlled clinical and animal studies published since the last systematic review together with the earlier case‐report literature. This review addresses the following question: do autologous platelet concentrates reduce inflammatory and replacement (ankylotic) root resorption and improve periodontal and pulpal healing outcomes, relative to no adjunct, after delayed or intentional tooth replantation, and does residual PDL viability at the time of application change this effect? The second, more specific part of this question is taken up directly in the Comparison and Discussion sections below, since it recurs as a testable distinction across the preclinical literature reviewed.

This question, in turn, spans two clinical scenarios that share a common biological substrate but differ in etiology, patient population, and treatment intent. The two are kept explicitly distinct throughout this review rather than treated as interchangeable. The first, and numerically dominant, scenario is delayed replantation after traumatic avulsion, in which an otherwise healthy tooth is lost from its socket by trauma, undergoes an uncontrolled extra‐oral dry period, and is replanted with the aim of preserving a tooth whose periodontal apparatus was previously sound. The preclinical animal and in vitro literature reviewed below almost exclusively model this scenario. The second is intentional replantation (IR) of a periodontally “hopeless” tooth, a planned surgical procedure in which a tooth already compromised by chronic periodontal disease is deliberately extracted, its root surface biomodified, and the tooth replanted as a last‐resort alternative to extraction. Parlak et al. is, to date, the only controlled clinical study of platelet concentrates in this second scenario, and it necessarily also reflects the confounding effect of concurrent root‐surface conditioning, which has no counterpart in the trauma literature. Because the extra‐oral time course, the baseline health of the periodontium, and the treatment goal differ so substantially between these two scenarios, findings from one are not assumed to generalize to the other anywhere in this review, and each is flagged explicitly where discussed.

## 2 Search Strategy and Study Selection

PubMed, Google Scholar, the Cochrane Library, Embase, Scopus, and Web of Science were searched from January to May 2026 (final search date: 31 May 2026) to identify studies on platelet concentrates in tooth avulsion and replantation. Search terms combined “platelet concentrates,” “PRP,” “PRF,” “delayed replantation,” and “periodontal ligament regeneration” using Boolean operators. Reference lists of the retrieved articles and of the systematic review by Khurshid et al. were hand‐searched for further studies (Khurshid et al. 2022).

Search terms were adapted to each database's controlled vocabulary (MeSH for PubMed, Emtree for Embase) and combined with the free‐text terms above; Cochrane Library, Scopus, Web of Science, and Google Scholar were searched with the corresponding free‐text combination, since these platforms do not support MeSH/Emtree mapping. Full database‐specific search strings are available from the corresponding author on request.

Eligible studies included in vivo animal studies, in vitro studies, case reports and case series, and comparative clinical studies, retrospective or prospective, evaluating any platelet concentrate (PRP, L‐PRP, PRF, or derivatives) in tooth avulsion, delayed replantation, or IR. No minimum follow‐up duration was set. Studies were restricted to English‐language publications; gray literature and unpublished studies were excluded. Both authors reviewed the retrieved records against these criteria and reached the final study set by discussion and consensus.

This narrative synthesis follows the SANRA scale (Baethge et al. 2019) and Grant and Booth's typology of a narrative review with a systematic search component (Grant and Booth 2009). No PRISMA protocol was registered, and no formal risk‐of‐bias tool was applied beyond the PRICE/PRIDE quality appraisal already reported by Khurshid et al. (2022).

## 3 Biology of PDL Healing After Tooth Avulsion

The PDL is a specialized connective tissue, richly vascularized and innervated, anchoring the root cementum to the inner wall of the alveolar bone socket (Polimeni et al. 2006). Tooth avulsion mechanically breaks the continuity of the PDL, tearing neurovascular bundles and leaving thin layers of soft tissue on both the root surface and the socket wall (Galler et al. 2021; Polimeni et al. 2006).

Physiological healing of the PDL after immediate replantation depends on whether these remnant PDL cells survive (Polimeni et al. 2006). When a tooth is replanted within a minimal extra‐oral period, viable progenitor cells within the PDL can proliferate and rebuild collagenous attachment fibers (Polimeni et al. 2006; Nasjleti et al. 1986). Successful primary healing follows a fairly distinct sequence (Polimeni et al. 2006). Hemostasis and clot formation come first, with intravascular clotting and extravascular fibrin deposition sealing injured vessels almost at once (Polimeni et al. 2006). An inflammatory phase follows, in which neutrophils and macrophages infiltrate the wound site within 24–48 h to clear cell debris and local bacteria (Polimeni et al. 2006). Fibroblasts, endothelial progenitor cells, and cementoblasts then migrate into the wound under paracrine signaling during a proliferative phase (Polimeni et al. 2006). A remodeling phase completes the sequence, in which Sharpey's fibers insert functionally into newly formed cementum and alveolar bone, restoring the physiological PDL width and its anti‐ankylotic barrier (Galler et al. 2021; Polimeni et al. 2006).

## 4 Pathophysiology of Delayed Tooth Replantation

When an avulsed tooth undergoes delayed replantation, with an extra‐oral dry time beyond 30–60 min, the biological cascade shifts away from primary tissue repair and towards pathological root resorption (Kostka et al. 2014; Galler et al. 2021).

Prolonged dry conditions cause desiccation and severe hypoxia, leading to diffuse necrosis of the attached PDL cells and pre‐cementoblasts lining the dentin surface (Galler et al. 2021). Once replanted into the host socket, this damaged, non‐viable organic layer sets off a marked immune response (Galler et al. 2021; Polimeni et al. 2006). In inflammatory root resorption, necrotic tissue or bacterial toxins from an infected root‐canal system pass through dentinal tubules, recruiting multinucleated clastic cells (dentinoclasts) that resorb cementum and dentin and leave deep, irregular resorption lacunae along the root (Galler et al. 2021). In replacement resorption (ankylosis), the loss of viable cementoblasts and an intact PDL cell lining removes the physiological barrier separating bone from dentin. Osteoblasts from the adjacent alveolar bone then invade the defect and deposit bone matrix directly onto the denuded root surface. Continuous remodeling gradually resorbs tooth structure and replaces it with bone, eventually causing infra‐position, loss of mobility, and tooth loss (Galler et al. 2021; Polimeni et al. 2006).

## 5 Biological Properties of Platelet Concentrates

Autologous platelet concentrates are biological formulations derived from centrifuged whole blood, grouped into distinct generations by their preparation protocol, architecture, and leukocyte content (Miron et al. 2026).

## 6 First‐Generation: Platelet‐Rich Plasma and Leukocyte‐ and Platelet‐Rich Plasma

First‐generation concentrates rely on two‐step centrifugation together with exogenous anticoagulants (e.g., sodium citrate) and activation agents (e.g., bovine thrombin or calcium chloride) to trigger clotting (Miron et al. 2026; Reichert da Silva Assunção et al. 2011). L‐PRP carries higher concentrations of both platelets and leukocytes; its leukocyte fraction has inherent antibacterial and immunomodulatory properties through phagocytosis and enzyme release, thought to help lower local bacterial load in contaminated sites (Miron et al. 2026).

## 7 Second‐Generation: PRF and Derived Matrices

Second‐generation concentrates, such as PRF (including Advanced PRF [A‐PRF] and Leukocyte‐PRF [L‐PRF]), are produced without anticoagulants or other additives (Miron et al. 2026; Dohan Ehrenfest et al. 2009). Centrifuging unmanipulated whole blood sets off natural coagulation, producing a dense, three‐dimensional fibrin meshwork that physically traps platelets, leukocytes, and key plasma proteins, as first described in the original classification of platelet concentrates by Dohan Ehrenfest et al. (2009). This intact fibrin scaffold reportedly sustains growth‐factor release for roughly 10–28 days, much longer than the rapid, largely exhausted release from PRP within the first day—a kinetic difference that may explain some of the divergent outcomes reported between first‐ and second‐generation concentrates in the replantation literature (Miron et al. 2026; Dohan Ehrenfest et al. 2009; Behnaz et al. 2021; Yang et al. 2018).

Beyond replantation, autologous platelet concentrates have also been studied as adjuncts in regenerative endodontic procedures for immature, necrotic permanent teeth, a parallel field with its own separately reported evidence on apical closure and continued root maturation (Miron et al. 2026). PRF protocols have more recently been extended to adjunctive dentoalveolar procedures such as tooth autotransplantation (Gjurčeski and Veleska‐Stevkovska 2024). The present review focuses on evidence concerning replanted, avulsed, or intentionally replanted teeth specifically.

## 8 Bioactive Cytokine Profile

Platelet concentrates store and gradually release high concentrations of key anabolic growth factors (Miron et al. 2026). Platelet‐derived growth factor (PDGF) stimulates mitogenesis and chemotaxis of mesenchymal stem cells, fibroblasts, and osteoblasts (Miron et al. 2026; Noda et al. 2012; Komatsu et al. 2022). Transforming growth factor‐β (β1/β2) (TGF‐β) drives extracellular matrix synthesis, promotes collagen production, and modulates cell differentiation (Polimeni et al. 2006; Miron et al. 2026). Vascular endothelial growth factor (VEGF) drives neo‐angiogenesis and revascularisation in ischemic tissue, while insulin‐like growth factor‐1 (IGF‐1) together with epidermal growth factor (EGF) supports cell survival, proliferation, and tissue repair (Polimeni et al. 2006; Miron et al. 2026).

## 9 Mechanisms of Action in Periodontal and Pulpal Regeneration

Platelet concentrates alter the local cellular environment during wound healing after tooth replantation through several linked mechanisms (Miron et al. 2026). During cellular proliferation and differentiation, growth factors such as PDGF and TGF‐β activate signal transduction pathways (e.g., ERK and MAPK) in PDL cells, osteoblasts, and dental pulp stem cells (DPSCs), driving cell‐cycle progression and raising proliferative markers such as Ki‐67; as proliferation subsides, these factors upregulate Nestin, an intermediate filament protein characteristic of functional odontoblasts and neural progenitor populations, together with alkaline phosphatase, encouraging differentiation into functional matrix‐secreting cells (Miron et al. 2026; Quispe‐Salcedo et al. 2024). In scaffolding and angiogenesis, the three‐dimensional fibrin framework serves as an autologous biopolymeric matrix that endothelial cells invade under the influence of released VEGF, forming capillary structures that restore blood supply to ischemic sockets and pulpal chambers (Miron et al. 2026; Navarro et al. 2019). Under the ischemic conditions caused by extended extra‐oral time, platelet concentrates have also been proposed to promote autophagy and cell‐survival pathways in dental pulp and PDL cells, which may reduce hypoxia‐induced apoptosis (Miron et al. 2026; Quispe‐Salcedo et al. 2024).

## 10 Preclinical Evidence: Animal Studies

A growing body of animal‐model evidence supports the mechanistic pathways outlined above. In a murine molar replantation model, Quispe‐Salcedo et al. evaluated topical L‐PRP application after immediate (1‐min) and intentionally delayed (30‐min) extra‐oral immersion (Quispe‐Salcedo et al. 2024). Histological and immunohistochemical analysis showed that L‐PRP reduced pulpal inflammation and promoted blood‐vessel reorganization; in immediate replantation, L‐PRP significantly accelerated hard‐tissue deposition within the pulp chamber at week 2 (*p* < 0.002) compared with controls, and under hypoxic conditions, it maintained pulpal repair dynamics and induced continuous Nestin immunoreactivity (Quispe‐Salcedo et al. 2024).

To assess the effect of PRF on severe avulsion injuries, Behnaz et al. used a canine model with mature, endodontically obturated teeth subjected to a 60‐min extra‐oral dry time (Behnaz et al. 2021). Sockets treated with PRF showed significantly less inflammatory root resorption than untreated control sockets (*p* = 0.031). PRF did not, however, significantly reduce replacement resorption (ankylosis) (*p* = 0.563), nor did it significantly change connective‐tissue fiber orientation or overall bone formation (Behnaz et al. 2021). A broadly similar split between inflammatory‐ and replacement‐resorption outcomes has been reported with first‐generation concentrates: in a canine model in which PDL and cementum were deliberately removed from the root surface before replantation, Yang et al. found that intracanal PRP significantly reduced ankylosis and promoted formation of PDL‐ and cementum‐like tissue relative to saline‐treated controls (Yang et al. 2018). The Yang et al. model removed the PDL and cementum entirely, whereas Behnaz et al. modeled a clinically realistic 60‐min dry time with the native root surface intact, so the two studies are not directly comparable (Behnaz et al. 2021; Yang et al. 2018). Taken together, though, they raise the possibility that the capacity of platelet concentrates to prevent ankylosis depends, at least in part, on the extent of residual PDL viability rather than being purely an intrinsic, generation‐independent property of PRP or PRF (Behnaz et al. 2021; Yang et al. 2018).

Because platelet concentrates deliver much of their biological effect through PDGF‐BB, studies using the isolated recombinant growth factor offer independent mechanistic support for this literature (Miron et al. 2026). In a canine model of partially denuded root replantation, Noda et al. found that intracanal PDGF‐BB produced newly organized PDL and significantly less replacement resorption than untreated controls (Noda et al. 2012). More recently, in a rat replantation model, Komatsu et al. showed that PDGF‐BB pretreatment markedly reduced ankylosis and restored functionally organized PDL collagen fibers, with the healing ligament regaining about 76% of the mechanical strength of non‐replanted teeth by 3 weeks (Komatsu et al. 2022). Neither study applied a platelet concentrate directly, but both indicate that PDGF‐BB alone can reproduce several of the anti‐ankylotic effects attributed to PRP and PRF, consistent with a PDGF‐driven mechanism of action (Noda et al. 2012; Komatsu et al. 2022).

In a within‐animal comparison of several first‐generation formulations, Assunção et al. replanted 64 premolars in adult dogs and found that only PRP activated with both calcium chloride and thrombin prevented root resorption; platelet‐poor plasma, calcium‐activated PRP alone, and a combination of bone‐marrow mononuclear cells with PRP all showed resorption, which indicates that the activation protocol, not merely the presence of platelets, shapes the biological outcome (Reichert da Silva Assunção et al. 2011). In a combined in vitro and canine study, Zhao et al. found that PRF granules combined with cell‐sheet fragments of PDL stem cells produced greater PDL cell proliferation and better histological bone and PDL regeneration than either PRF or PDL stem cells alone, which points to cell‐based co‐delivery as a promising direction for future preclinical work (Zhao et al. 2013).

Most recently, Li et al. developed a delayed‐replantation model in immature permanent teeth using eight experimental beagle dogs and two untreated controls, in which a liquid/gel mixed‐phase concentrated growth factor preparation applied to extracted mandibular canines improved both periodontal and pulpal healing relative to untreated controls, extending the preclinical evidence for platelet concentrates to actively developing roots (Li et al. 2026).

## 11 In Vitro Evidence

Alongside the in vivo data, in vitro work has examined the direct effect of platelet concentrates on PDL cells exposed to extended dry time (Navarro et al. 2019; Hiremath et al. 2014). Hiremath et al. treated PDL cells attached to extracted human teeth with PRF combined with platelet‐poor plasma after 40 min of drying and found higher PDL cell proliferation than with platelet‐poor plasma alone or no treatment, supporting at the cellular level the biological plausibility of the proliferative effects described above, free of the confounds inherent to whole‐animal models (Hiremath et al. 2014). Navarro et al. assessed PDL cell viability after exposure to PRF lysate and reported improved maintenance and recovery of PDL cell viability in vitro, lending further mechanistic support to the same proliferative effects (Navarro et al. 2019).

## 12 Clinical Evidence in Human Studies

In a retrospective comparative study, Parlak et al. evaluated the clinical efficacy of combining PRF with IR in 32 periodontally compromised, “hopeless” mandibular anterior teeth (Parlak et al. 2026), a distinct clinical scenario from the traumatic‐avulsion model underlying the preclinical evidence discussed above: these are periodontally diseased teeth deliberately extracted and replanted as a planned procedure, with tetracycline‐HCl root conditioning applied to both study arms, rather than teeth avulsed by trauma and replanted after an uncontrolled extra‐oral period. This difference in etiology, baseline periodontal health, and concurrent root‐surface treatment should be borne in mind when relating its findings to the trauma literature reviewed elsewhere in this article. Sockets in the test group received an autologous PRF membrane at the base before replantation (Parlak et al. 2026). Over a 15‐month follow‐up, the IR + PRF group showed significantly greater clinical attachment level (CAL) gains at the mesial site (4.43 vs 1.79 mm, *p* = 0.004) and the midlingual site (3.20 vs. 0.94 mm, *p* = 0.018), along with significantly greater probing‐depth (PD) reductions at the mesial (*p* = 0.043) and midlingual (*p* = 0.017) sites, and no clinical or radiographic sign of ankylosis or root resorption in either group over this short‐term follow‐up (Parlak et al. 2026). This is a single retrospective, non‐randomized, unblinded comparison with a modest sample. Multiple site‐level statistical tests were reported without a stated multiplicity correction, and the original study reports point estimates and *p*‐values without confidence intervals.

This controlled comparative design marks a methodological step forward from the case‐report literature that had, until recently, made up the entire clinical evidence base for platelet concentrates in intentional and delayed replantation (Khurshid et al. 2022; Parlak et al. 2026). Ten such case reports, each describing a single replanted tooth, appear in the only systematic review conducted on this topic (Khurshid et al. 2022). Among them, Tözüm et al. and Demir et al. each intentionally replanted a periodontally hopeless mandibular incisor after applying PRP to the socket, reporting reduced mobility and radiographic bone fill, outcomes directionally consistent with, though far less rigorously controlled than, the improvements later reported by Parlak et al. for the same clinical indication (Parlak et al. 2026; Tözüm et al. 2006; Demir et al. 2007). In cases of delayed replantation after traumatic avulsion, Priya et al. applied intracanal PRP more than 8 h after extra‐oral dry time and reported complete resolution of periapical radiolucency and normal mobility at 12 months, while Johns et al. reported a positive pulp‐sensibility test at 24 months after intracanal PRF was used following an 8‐h extra‐oral storage period in milk (Priya et al. 2016; Johns et al. 2013). In a case involving a necrotic, immature second premolar, Torabinejad and Turman combined PRP with mineral trioxide aggregate intracanally and observed continued root development, apical closure, and return of pulp sensitivity within 5.5 months, suggesting that platelet concentrates may support pulpal as well as periodontal regeneration in select cases (Torabinejad and Turman 2011).

Beyond the studies captured in the systematic review by Khurshid et al. several more recent reports describe favorable outcomes with second‐generation concentrates in delayed replantation (Khurshid et al. 2022). Suresh used injectable PRF (i‐PRF) in the delayed replantation of an avulsed permanent tooth with an extended extra‐oral dry time, reporting favorable soft‐tissue healing at follow‐up (Suresh 2021). Yang et al. likewise applied autologous PRF in two cases of delayed replantation of “hopeless” avulsed teeth, observing satisfactory periodontal healing over 12 months without progressive resorption (Yang et al. 2023). In a case involving multiple avulsed mandibular anterior teeth, Mareddy et al. reported favorable outcomes with advanced PRF over a 36‐month follow‐up, one of the longest reported for this clinical indication (Mareddy et al. 2026).

Across this case‐report literature, outcomes were consistently favorable, and no adverse events attributable to the platelet concentrate itself were reported (Tözüm et al. 2006; Demir et al. 2007; Priya et al. 2016; Johns et al. 2013; Suresh 2021; Yang et al. 2023; Mareddy et al. 2026). Each report, however, described a single patient with no comparator group, radiographic rather than histological confirmation of healing, and follow‐up generally under 2 years. This consistency must be weighed against a substantial risk of publication bias and an inability to rule out spontaneous healing or the effect of concurrent endodontic and periodontal therapy (Khurshid et al. 2022; Nagendrababu et al. 2019). Parlak et al. fill an important gap by providing the first study with a contemporaneous internal control group for this clinical indication, even though it too remains retrospective and non‐randomized (Parlak et al. 2026).

## 13 Comparison Between Different Platelet Concentrates

PRP and L‐PRP require multi‐step centrifugation with anticoagulants and clotting activators, producing a liquid/gel formulation with a rapid burst release of growth factors over 1–3 days (Miron et al. 2026; Reichert da Silva Assunção et al. 2011). PRF, L‐PRF, and A‐PRF, by contrast, are prepared without additives, forming a flexible three‐dimensional fibrin matrix that releases cytokines gradually over roughly 10–28 days and acts both as a bio‐scaffold and a growth‐factor reservoir (Miron et al. 2025; Gjurčeski and Veleska‐Stevkovska 2024). The animal evidence summarized above suggests that this difference in release kinetics and matrix architecture, together with the degree of activation (e.g., calcium‐ vs*.* thrombin‐activation of PRP) (Reichert da Silva Assunção et al. 2011) and the extent of residual PDL viability at the time of application (Behnaz et al. 2021; Yang et al. 2018), determines biological effect more consistently than the concentrate's generation on its own.

## 14 Protocol Optimization and the Evolution of PRF Preparation

Beyond the differences between platelet‐concentrate generations discussed above, the preparation of PRF itself has continued to evolve since Choukroun's original fixed‐angle protocol. The low‐speed centrifugation concept, introduced to limit mechanical trauma to platelets and leukocytes, favors horizontal (swing‐out) over fixed‐angle rotors, since horizontal centrifugation redistributes cellular components more evenly across the fibrin clot and yields higher platelet and leukocyte recovery at an equivalent relative centrifugal force (RCF) (Miron et al. 2024). Building on this principle, concentrated growth factor and concentrated‐PRF (C‐PRF) protocols centrifuge whole blood at higher RCFs before harvesting only the platelet‐ and leukocyte‐rich buffy‐coat layer, yielding a liquid concentrate with a substantially higher cellular payload than conventional low‐speed liquid PRF (i‐PRF) (Li et al. 2026; Miron et al. 2024). Of the replantation studies summarized in Table 1, only Li et al. explicitly used a concentrated, liquid/gel mixed‐phase growth‐factor preparation. None of the others reported whether a fixed‐angle or horizontal rotor was used. It therefore remains unknown whether the modest and sometimes inconsistent effects reported for first‐ and early second‐generation concentrates in delayed replantation would be reproduced, or exceeded, with an optimized, horizontally centrifuged, high‐yield preparation.

**Table 1:** Scientific evidence synthesis matrix.

Study	Design and sample	Intervention and outcomes assessed	Key findings	Limitations
Parlak et al. (2026)	Retrospective comparative study; 32 mandibular anterior teeth, 32 patients (15 IR + PRF, 17 IR control); 15‐month follow‐up	Intentional replantation (IR) of hopeless teeth (≥ 60% bone loss); tetracycline‐HCl root conditioning, MTA apical seal; PRF at socket base (test group); splinted 6 months. Outcomes: PD, CAL, PI, GI, KTH, survival, ankylosis, resorption	Significantly greater CAL gain with PRF at mesial (4.43 vs. 1.79 mm, *p* = 0.004) and midlingual (3.20 vs. 0.94 mm, *p* = 0.018) sites; greater PD reduction at both sites; no ankylosis or resorption in either group	Retrospective, non‐randomized, unblinded; no histological confirmation of true attachment versus long junctional epithelium; confounded by root biomodification
Quispe‐Salcedo et al. (2024)	In vivo mouse study; maxillary first molars, 40 male CD1 ICR mice; sacrifice day 1–week 4	Immediate (1‐min) versus delayed (30‐min) extra‐oral immersion; socket treated with 1.5 µL L‐PRP or untreated. Outcomes: pulpal histology (H&E, AZAN), Nestin/Ki‐67 immunohistochemistry	l‐PRP enhanced pulpal healing and accelerated hard‐tissue deposition at week 2 (*p* < 0.002); Ki‐67 proliferation was maintained early but reduced by week 2 in delayed group; attenuated hypoxic injury	Murine molar versus human model; small socket volume; donor variability; no splinting or endodontic intervention; pulpal focus only
Behnaz et al. (2021)	In vivo randomized split‐mouth study; 16 mature teeth, 2 beagle dogs; 8‐week follow‐up	Delayed replantation (60‐min dry time) with endodontic obturation; socket filled with PRF or untreated. Outcomes: cementum repair, bone formation, resorption/ankylosis, PDL fiber orientation	Significantly lower inflammatory root resorption with PRF (*p* = 0.031); no significant difference in ankylosis (*p* = 0.563), bone formation, or fiber alignment	Small sample (2 animals, 16 teeth); short follow‐up; mature root‐filled model precluded pulpal assessment; 60‐min dry time may exceed PRF's capacity
Torabinejad and Turman (2011)	Case report; 1 patient (11 years); maxillary second premolar, open apex	Accidental avulsion with pulpal necrosis; PRP + MTA intracanally, antibiotic paste, root canal therapy. Outcomes: radiographic healing, pulp sensibility	Continued root development, apical closure, resolution of periapical radiolucency; pulp sensibility returned at 5.5 months	Single‐patient design; short follow‐up; no confirmation that sensibility reflected true revascularisation
Yang et al. (2018)	In vivo histologic/histomorphometric study; 36 premolar/incisor roots, 6 mongrel dogs	PDL and cementum removal, then intracanal PRP versus saline (PDL/cementum intact or removed). Outcomes: ankylosis, PDL‐/cementum‐like tissue formation	PRP significantly reduced ankylosis and promoted PDL‐/cementum‐like tissue versus saline‐treated, denuded controls	Small canine cohort; blinding not reported; 8‐week follow‐up only; artificially denuded roots may not reflect clinical avulsion
Zhao et al. (2013)	Combined in vitro (human PDLSC) and in vivo (dog) study; 36 incisors, 6 dogs; 8‐week follow‐up	Cell‐sheet PDLSC fragments + PRF granules versus PRF alone, PDLSCs alone, or none. Outcomes: ALP/BSP/OCN/Col‐1 expression; histological bone/PDL regeneration	PRF + PDLSCs induced greater proliferation and superior bone/PDL regeneration than either component alone	Small animal cohort; in vitro‐to‐in vivo translation unconfirmed clinically; technique not readily standardized
Khurshid et al. (2022)	Systematic review; 10 case reports, 3 animal studies, 1 combined animal/in vitro study, 1 in vitro study; PRISMA search of 4 databases	Narrative synthesis of PRP/PRF in tooth replantation; quality‐appraised with PRICE (case reports) and PRIDE (animal/in vitro) tools	Majority reported improved periapical bone regeneration, reduced mobility, and favorable healing; no meta‐analysis feasible	High risk of bias in most studies; heterogeneous protocols and inadequate sample sizes to establish efficacy

*Note:* Table summarizes the study design, intervention, principal outcomes, and reported limitations of the key studies discussed in this review.

Abbreviations: ALP, alkaline phosphatase; BSP, bone sialoprotein; CAL, clinical attachment level; Col‐1, type I collagen; GI, gingival index; IR, intentional replantation; KTH, keratinized tissue height; MTA, mineral trioxide aggregate; OCN, osteocalcin; PD, probing depth; PDLSC, periodontal ligament stem cell; PI, plaque index.

Collection‐tube material is a further, often overlooked, source of variability. Silica‐coated plastic tubes activate coagulation via silica microparticles that become incorporated into the fibrin matrix and have themselves been reported as a possible biological contaminant, whereas plain glass tubes avoid this risk but can yield different clot dimensions and growth‐factor release kinetics from an otherwise identical centrifugation protocol (Miron et al. 2021). Because tube type interacts with rotor geometry to determine the cellular architecture of the final clot, describing PRF only as an anticoagulant‐free preparation, without also specifying tube material, is insufficient to reproduce a given protocol. Wider adoption of standardized nomenclature and step‐by‐step instructions for L‐PRF preparation and clinical application, recently formalized for other regenerative indications, would allow the tooth‐replantation literature to converge on directly comparable, reproducible protocols (Quirynen, Blanco, et al. 2025).

## 15 Current Limitations of the Evidence

Several limitations restrict the strength of conclusions that can be drawn at present (Khurshid et al. 2022; Nagendrababu et al. 2019, 2020). Human clinical trials, including the recent comparative study by Parlak et al. and the earlier case‐report literature, are limited to non‐invasive clinical parameters (PD, CAL) and radiography, so it remains impossible to confirm whether clinical attachment gain reflects true physiological PDL regeneration or soft‐tissue repair through a long junctional epithelium (Khurshid et al. 2022; Parlak et al. 2026). Methodological diversity in centrifugation speed, RCF, spin duration, tube type, and activation protocol also produces wide variability in growth‐factor concentrations across studies, as the divergent within‐study results for differently activated PRP formulations illustrate (Miron et al. 2026; Reichert da Silva Assunção et al. 2011). This problem is compounded because none of the replantation studies reviewed above report centrifugation parameters in a fully standardized format: rotor design (fixed‐angle vs*.* horizontal/swing‐out), tube angulation, radius, and collection‐tube material are seldom stated alongside the RCF, even though RCF calculated at the rotor minimum, at the clot, or at the maximum radius can differ substantially for an identical revolutions‐per‐minute (RPM) setting, so RPM values alone cannot be compared meaningfully across studies (Miron et al. 2019; Herrera‐Vizcaino 2023). Following the reporting standard proposed by Miron et al. and the AR^2^T^3^ framework (Angle, Radius, RCF, Time, Tube size, and Tube material), future replantation studies should state RCF‐max explicitly rather than RPM alone, together with rotor angulation and tube type, to allow meaningful cross‐study comparison and pooling (Miron et al. 2019; Herrera‐Vizcaino 2023). Translating findings from animal models is further complicated by differences in animal size, root‐canal treatment, splinting protocols, and follow‐up duration across studies (Behnaz et al. 2021; Yang et al. 2018; Li et al. 2026). A formal quality appraisal of the broader replantation literature, using the PRICE guidelines for case reports and the PRIDE guidelines for animal and in vitro studies, found that most case reports lacked a comparator and that only a minority of animal studies used blinding or reported animal or tooth attrition during the experiment, which rules out meta‐analysis and keeps the overall evidence at a qualitative, hypothesis‐generating level (Khurshid et al. 2022; Nagendrababu et al. 2019, 2020). Wider use of these reporting frameworks by future investigators, together with prospective and, where ethically feasible, randomized designs, would meaningfully strengthen the evidence base (Nagendrababu et al. 2019, 2020).

## 16 Limitations of This Review

Beyond the limitations of the primary evidence described above, this review is itself narrative rather than systematic in design, and so carries the selection and reporting biases inherent to that format.

The evidence base also leans heavily on a small number of preclinical models and a single recent comparative clinical study (Parlak et al. 2026), so the conclusions drawn here should be read as hypothesis‐generating rather than definitive until confirmatory randomized trials are available.

## 17 Conclusion

Platelet concentrates, including PRP, L‐PRP, and PRF, are biologically active autologous biomaterials that support tissue healing in dental traumatology and IR (Miron et al. 2026). Evidence spanning in vitro (Navarro et al. 2019; Hiremath et al. 2014), animal (Behnaz et al. 2021; Quispe‐Salcedo et al. 2024), and one controlled retrospective clinical study indicates that platelet concentrates promote pulpal healing and tertiary dentin formation (Quispe‐Salcedo et al. 2024), reduce inflammatory root resorption (Behnaz et al. 2021), and support periodontal cell viability (Navarro et al. 2019; Hiremath et al. 2014), while two decades of case‐report literature further support favorable periodontal, and in some instances pulpal, outcomes in individual patients (Khurshid et al. 2022). In one retrospective comparative clinical study, combining PRF with IR protocols was associated with greater soft‐tissue attachment gain and reduced probing depths, though this finding awaits confirmation in prospective, randomized research (Parlak et al. 2026). Platelet concentrates alone, however, appear insufficient to prevent replacement resorption or ankylosis after severe extra‐oral desiccation, and the degree of residual PDL viability, rather than the concentrate's generation alone, may set this ceiling on effect (Behnaz et al. 2021; Yang et al. 2018). These findings should be weighed alongside current International Association of Dental Traumatology (IADT) guidance for the management of tooth avulsion, which does not at present include platelet concentrates as a recommended adjunct; the evidence summarized here is best regarded as an emerging, investigational adjunct rather than a change to established practice (Fouad et al. 2020). Further standardized preclinical research, adherence to PRICE/PRIDE reporting frameworks, and prospective randomized clinical trials are needed to settle the role of platelet concentrates in PDL regeneration (Nagendrababu et al. 2019, 2020).

## 18 Clinical Relevance

Platelet concentrates (PRP, L‐PRP, PRF) are biologically plausible, low‐cost autologous adjuncts that can improve pulpal healing and reduce inflammatory root resorption after tooth avulsion and replantation (Miron et al. 2026; Behnaz et al. 2021; Quispe‐Salcedo et al. 2024).

Current evidence indicates that platelet concentrates alone do not reliably prevent replacement resorption (ankylosis) once PDL viability is severely compromised, which should temper how prognosis is discussed with patients after delayed replantation (Behnaz et al. 2021; Yang et al. 2018).

Combining PRF with IR offers the strongest clinical evidence available for periodontally hopeless teeth, but this rests on a single retrospective comparative study together with a preclinical, small‐scale, or case‐report evidence base, and calls for cautious, individualized use pending randomized trials (Khurshid et al. 2022; Parlak et al. 2026).

Current IADT guidance for the management of tooth avulsion does not include platelet concentrates as a recommended adjunct; clinicians should treat the evidence summarized here as investigational rather than as part of established practice (Fouad et al. 2020).

## Author Contributions

**Walaa Almarji:** literature search, data curation, writing – original draft. **Mohannad Laflouf:** conceptualization, supervision, writing – review and editing. Both authors approved the final manuscript.

## Funding

The authors have nothing to report.

## Ethics Statement

This article is a narrative review of previously published literature; the authors collected no original human or animal data, so no institutional ethics or animal‐welfare approval was required for this work.

## Consent

No patient‐identifiable data, images, or original case material are presented in this review.

## Conflicts of Interest

The authors declare no conflicts of interest.

## Data Availability

No new data were generated or analyzed for this review. All data discussed are available in the cited publications listed in the References section.
